# The *Onchocerca volvulus* Cysteine Proteinase Inhibitor, *Ov*-CPI-2, Is a Target of Protective Antibody Response That Increases with Age

**DOI:** 10.1371/journal.pntd.0000800

**Published:** 2010-08-24

**Authors:** Fidelis Cho-Ngwa, Jing Liu, Sara Lustigman

**Affiliations:** 1 Laboratory of Molecular Parasitology, Lindsley F. Kimball Research Institute, New York Blood Center, New York, New York, United States of America; 2 Department of Biochemistry and Microbiology, Faculty of Science, University of Buea, Buea, Cameroon; Michigan State University, United States of America

## Abstract

**Background:**

Despite considerable efforts, a suitable vaccine against *Onchocerca volvulus* infection has remained elusive. Herein, we report on the use of molecular tools to identify and characterize *O. volvulus* antigens that are possibly associated with the development of concomitant immunity in onchocerciasis.

**Methodology/Principal Findings:**

Third-stage larvae (L3) and molting L3 (mL3) *O. volvulus* stage-specific cDNA libraries were screened with a pool of sera from chronically infected patients who had likely developed such immunity. The 87 immunoreactive clones isolated were grouped into 20 distinct proteins of which 12 had already been cloned and/or characterized before and 4 had been proven to be protective in a small *O. volvulus* animal model. One of these, onchocystatin (*Ov*-CPI-2), a previously characterized *O. volvulus* cysteine proteinase inhibitor was, overall, the most abundant clone recognized by the immune sera in both the L3 and mL3 cDNA libraries. To further characterize its association with protective immunity, we measured the IgG subclass and IgE class specific responses to the antigen in putatively immune (PI) and infected (INF) individuals living in a hyperendemic area in Cameroon. It appeared that both groups had similar IgG3 and IgE responses to the antigen, but the INF had significantly higher IgG1 and IgG4 responses than the PI individuals (*p*<0.05). In the INF group, the IgG3 levels increased significantly with the age of the infected individuals (r = 0.241; *p*<0.01). The IgG1 responses in the INF were high regardless of age. Notably, culturing L3 *in vitro* in the presence of anti-*Ov*-CPI-2 monospecific human antibodies and naïve neutrophils resulted in almost complete inhibition of molting of L3 to L4 and to cytotoxicity to the larvae.

**Conclusions/Significance:**

These results add to the knowledge of protective immunity in onchocerciasis and support the possible involvement of anti-*Ov*-CPI-2 IgG1 and/or IgG3 cytophilic antibodies in the development of protective immunity in the PI and the INF. The results further support the consideration of *Ov*-CPI-2 as a leading target for an anti-L3 vaccine.

## Author Summary

Onchocerciasis is a chronic and highly debilitating disease of humans caused by a worm called *Onchocerca volvulus*. This worm can live in the human body for over 15 years. The disease affects mainly the skin and eyes and is the second leading infectious cause of blindness worldwide. There is currently no vaccine to prevent the infection. Available drugs can give short-term relief but cannot cure the infection. To prevent infection, a vaccine against the third-stage infective larva, L3, or the developing larva is required. These stages were shown to be the targets of protective immunity that develops in individuals who live in onchocerciasis endemic regions. One type of protective immunity has been shown to develop with age and is called concomitant immunity. In the present study, we have identified a number of larval antigens that may be associated with the development of such immunity. The most prominent of these antigens was *Ov*-CPI-2, also called onchocystatin, which had previously been shown to be a promising vaccine candidate. This antigen was further characterized and confirmed to be possibly also a target of immune protection that develops in the infected individuals with age and is referred to as concomitant immunity.

## Introduction

Human onchocerciasis (river blindness) is a highly debilitating and blinding disease caused by the nematode, *Onchocerca volvulus.* Over 37 million people in endemic countries of tropical Africa, Latin America and the Arabian Peninsula are infected [1]. Currently, there is no cure for the disease and a vaccine is yet to be developed. Importantly, protective immunity against *O. volvulus* larvae has now been definitively demonstrated in humans, cattle and mice, thereby proving the conceptual underpinnings that a vaccine can be produced against this infection [2]. Both clinical and epidemiological data support also the concept that acquired immunity against *O. volvulus* infection occurs in infected humans and that this immunity increases with age. In chronically infected (INF) individuals in highly endemic settings, the skin microfilariae (mf) density tends to increase with age until the ages of 20 to 40 in most studies, suggesting that older individuals do develop a means of limiting further infections [3]. A similar trend was also observed for adult worms where in areas of high transmission, the number of palpable nodules reached an average of 3–5 over time and then leveled-off [4]. While it is conceivable that products from the existing infections may as well prevent super infections from getting established, the conglomeration of at times a few adult worms in one nodule is rather at odds with this perception. The leveling off of patent infections with age is consistent with the concept of concomitant immunity [5], [6], which is characterized by the tendency of the host to eliminate the newly introduced infective-stage larvae (L3), while the established adult worms and microfilariae are left almost unaffected.

Further evidence for the development of concomitant immunity in filarial infections comes from studies of lymphatic filariasis, where it was associated with parasite stage-specific immune responses. Levels of antibodies against *Wuchereria bancrofti* infective stage larvae (L3) were found to increase with duration of exposure [7], and there were differences in the class and subclass antibody responses to adult versus larval antigens of *Brugia malayi* [8]. More recently, the concept of concomitant immunity was further verified experimentally using the *Acanthocheilonema vitae* jird model [9]. The L3 and the developing or molting L3 (mL3) have been the focus of vaccination studies in filarial infections [10]–[15].

The roles of the different antibody isotype and subclass responses to *O. volvulus* crude extract of larval proteins and defined protective recombinant proteins in onchocerciasis have been studied in greater detail. Importantly, analysis of the immunoglobulin class and subclass responses to some of the protective antigens revealed that these proteins induced mainly cytophilic antibodies, IgG1, IgG3 and/or IgE in PI and INF. Boyer and colleagues [16] showed that in areas of high transmission, high IgG3 responses are associated with protective immunity, a finding which was later confirmed by other studies [6], [17]. In our previous studies we have shown that IgG3 and IgE responses to crude extracts of L3 as well as the IgG1 and IgG3 responses to a highly abundant larvae-specific protein, *Ov*-ALT-1, were also associated with concomitant immunity [6]. The filarial *Ov*-ALT-1 family members have been shown to be protective against infection with L3 of *O. volvulus* and in lymphatic filariasis in small animal models [13], [18]–[20]. Although the precise mechanisms that mediate killing of *O. volvulus* L3 in the PI and in individuals who developed protective immunity with age are still unknown, data from many studies support the view that antibodies are part of the effector mechanisms against incoming *O. volvulus* larval infection and could, together with the Th1 and/or Th2 cytokines produced, induce efficient anti-L3 antibody-dependent cell mediated cytotoxicity (ADCC) reactions [6], [16], [21]. However, no studies were performed to specifically identify and clone larval proteins that are possibly associated with the development of concomitant immunity. In order to identify such proteins, we have screened L3 and mL3 *O. volvulus* stage-specific cDNA libraries with a pool of sera from chronically infected patients who have likely developed concomitant immunity. One of the antigens identified, *Ov*-CPI-2 (onchocystatin), a previously characterized *O. volvulus* cysteine proteinase inhibitor was the most abundant clone recognized by the immune sera, and was selected for further analysis in two groups of individuals: the INF and the putatively immune (PI) individuals [22]. Additionally, we present results on *in vitro* cytotoxic effects of human neutrophils on third-stage larvae of *Onchocerca volvulus* in the presence of mono-specific antibodies to *Ov*-CPI-2.

## Materials and Methods

### Study population and sera

The protocols used in this study were approved by the New York Blood Center's IRB and by an NIH accredited Institutional Review Board of the Medical Research Council Kumba, Cameroon. Each participant provided informed consent by signing or thumb printing a consent form after reading it or after the content of the form was read and/or explained to them. The serum samples were collected from residents of 5 villages around Kumba, Cameroon, a hyperendemic area for onchocerciasis [6]. These villages were Marumba I, Marumba II, Boa Bakundu, Bombanda, and Bombele. All participants were born or had resided for more than 10 years in the villages. The standard skin snip test for detection of microfilariae (mf) was performed on each subject and clinical symptoms of onchocerciasis were recorded. The averages of the mf counts of four skin snips taken from each individual were used in estimating the individual skin mf densities. None of the subjects had received ivermectin treatment prior to the collection of blood. In this study we used 176 serum samples collected from infected individuals; 115 males and 61 females, ranging in age from 3 to 75 years. In addition, serum samples of 21 putatively immune (PI) subjects were studied. These individuals had no signs or history of onchocerciasis and were parasitologically negative during at least a two-year follow-up survey employing the standard skin snip test and a polymerase chain reaction (PCR) assay [6], [22].

### Immunoscreening of *O. volvulus* cDNA libraries

In order to identify *O. volvulus* L3 and mL3 larval proteins that are recognized by sera from infected individuals who have likely developed concomitant immunity, λ-Unizap XR cDNA expression libraries of these two life cycle stages (kindly obtained from Dr. Steven Williams via the NIAID/NIH Filariasis Research Reagent Repository Center) were immunoscreened as previously described [12] with only slight modifications. A pool of sera was prepared using equal volumes of 17 serum samples from infected individuals, 35 years of age and above, who had previously been shown to have high anti-L3 and mL3 antibody titres [6]. The serum pool was first pre-cleared with *Escherichia coli* lysate (SIGMA, St Louis, MO) and then used at a final dilution of 1∶400 for the immunoscreening of the cDNA libraries, which were plated at 25,000 plaque-forming units (pfu) per 150 mm diameter plates. The secondary antibody, goat anti-human IgG conjugated to horseradish peroxidase (KPL, Gaithersburg, MD) was used at 1∶8000 dilution. The positive plaques were identified by incubating the processed plaque lifts in a solution of phosphate buffered saline (PBS) containing 0.67 mg/ml diaminobenzidine tetrahydrochloride (DAB) (SIGMA, St Louis, MO) and 1 µl/ml of 30% H_2_O_2_. All positive plaques were recovered and re-purified using one or two additional screening cycles. Single phage clones were eventually recovered and stored in standard phage buffer (SM buffer) in the presence of 0.3% chloroform.

### Polymerase chain reaction (PCR) and sequencing

The inserts of the positive phages were amplified using PCR with the M13 forward and M13 reverse primers. Briefly, 45 µl of PCR supermix (Invitrogen, Carlsbad, USA), 1 µl of each primer (100 ng/µl) and 1–10 µl of the eluted recombinant phage were mixed and incubated at 94°C for 5 minutes. Amplification was carried out for 35 cycles of: denaturation at 94°C for 45 seconds, annealing at 55°C for 45 seconds, and extension at 72°C for 75 seconds. The final amplification cycle included an additional extension step at 72°C for 10 minutes (Eppendorf MasterClycler Gradient, Eppendorf, USA). The PCR amplicons were analyzed by standard agarose gel electrophoresis on a 1.2% gel. Only clones that produced a single band on electrophoresis were selected for sequencing. Those with two or more bands were further plaque purified before sequencing. The PCR products were purified using the Rapid PCR Purification System kit (Marligen Biosciences, Ijamsville, MD) and quantified. Then 10–20 ng of each PCR product was sequenced using the M13 reverse universal sequencing primer (GENEWIZ DNA Sequencing Service, New Jersey, NJ). Gene specific primers were prepared to complete the sequencing reactions in both directions.

### Bioinformatics analyses

BLASTx and BLASTn searches were carried out using the cDNA sequences in frame with the EcoR1 cloning site at the 5′ terminus against the GenBank non-redundant protein and nucleotide databases, respectively, as well as against the Nembase2 and Wormbase databases. Open reading frames were computed using the DNASIS software package (Hitachi, CA) or the ORFing facility at the NCBI homepage (www.ncbi.nlm.nih.gov).

### Immune responses to recombinant *Ov*-CPI-2

Recombinant *Ov*-CPI-2 fused to *Schistosoma japonicum* glutathione *S*-transferase (GST) polypeptide was expressed in the pGEX-1N vector and purified as previously described [13]. The IgG1, IgG3, IgG4 and IgE responses to recombinant GST-*Ov*-CPI-2 were determined by indirect ELISA essentially as described by MacDonald *et al* [6]. Briefly, GST-*Ov*-CPI-2 or GST were diluted in 0.05M carbonate-bicarbonate buffer, pH 9.6 at a concentration 1 µg/ml and used to coat the wells of Immunolon 2 plates (Dynex, VA), overnight at 4°C. After blocking excess binding sites with 3% (w/v) casein, individual serum samples which had previously been pre-cleared with *E. coli* lysate were diluted to 1∶200 and reacted with the bound antigen for 90 minutes at room temperature. For the analysis of IgE levels, plates were coated with 10 µg/ml of rOv-CPI-2 or the control (GST) and serum samples were pre-absorbed with protein G-Sepharose (Pharmacia) before being used at a 1∶20 dilution. For IgG subclass responses, the bound antibodies were detected by using a 1∶1000 dilution of monoclonal antibodies against different human IgG subclasses (Hybridoma Reagent Laboratory, Kingsville, Md.). This step was followed by incubation with a 1∶8000 dilution of horseradish peroxidase-conjugated goat anti-mouse IgG (H+L) (KPL, Gaithersburg, MD) for another 60 minutes at room temperature. IgE antibodies in the sera were detected by using a horseradish peroxidase-conjugated, ε-chain-specific anti-human IgE monoclonal antibody (Zymed, San Francisco, CA) at a dilution of 1∶750. Tetramethylbenzidine (SIGMA, St Louis, MO) was prepared according to the supplier's recommendations and used as the enzyme substrate. Absorbance (OD) was read after stopping the reaction with an equal volume of 1M H_2_SO_4_ at 450 nm using an Emax ELISA reader (Molecular Devices, CA). The OD values for r*Ov*-CPI-2 are presented as the net values after subtracting the OD values of the GST control from those of the corresponding GST-*Ov*-CPI-2 readings for each serum sample.

### *In vitro* inhibition of L3 molting and killing assays

Monospecific human antibodies to recombinant *Ov*-CPI-2 were purified as described by Lustigman *et al.* [23]. Briefly, cells from induced lysogenic cultures of λgt11 expressing *Ov*-CPI-2 were resuspended in 0.1 M phosphate buffer containing 10 mM dithiothreitol (DTT) and sonicated. Sodium Dodecyl Sulphate (SDS) was added to a final concentration of 1% and then centrifuged at 10000× g for 10 min at 4°C. The supernatant was desalted on a G-25 column using 0.1 M phosphate buffer, 1 mM DTT, 0.1% SDS, pH 6.8. The supernatants containing β-galactosidase or β-galactosidase *Ov*-CPI-2 fusion polypetide were then coupled to CNBr-Sepharose 4B using the protocol provided by the manufacturer (Pharmacia). Antibodies from 100 ml of plasma of an infected individual, who had high titers of anti-*Ov*-CPI-2 antibodies, were affinity purified on the immobilized polypeptides. The mono-specificity of the eluted antibodies was confirmed by Western blot analysis and immunogold electron microcopy [23]. Negative antibodies were purified from the same donor sera from an affinity column containing crude extracts of induced lysogenic culture of λgt11 coupled to the CNBr beads. The IgG titer of the anti-*Ov*-CPI-2 purified antibodies was 1∶3125 as determined by ELISA, while the negative antibodies had an anti-*Ov*-CPI-2 cross reacting antibody response at 1∶5 dilution. The purified antibodies were passed through a 0.22 µM filter for sterilization.

The L3 killing or inhibition of molting assays were done as described by Johnson *et al*. [21]. Briefly, L3 were washed in RPMI-1640 with 1× GPS (glutamine, penicillin, streptomycin) and the worms were diluted to 5 worms per 50 µL in the medium containing 20% fetal calf serum (complete medium). Worms were distributed to 10 wells of a 96-well plate per treatment group and 2×10^5^ normal neutrophils isolated by dextran sedimentation were added to each well in 50 µL of complete medium. Then, 100 µL of the anti-*Ov*-CPI-2 purified antibodies or the negative control antibodies were added to each of the 10 wells. Complete medium without antibodies was also included as a control. The 96-well plates were then incubated at 37°C in a 5% CO_2_ incubator until day 6 when the cultures were observed for molting; the presence or absence of the fourth-stage larvae (L4) and the empty cast of the L3 under an inverted microscope. Viability of the larvae was then determined by MTT (3-(4,5 dimethylthiazol-2yl)-2,5 diphenyl tetrazolium bromide) staining as previously described [21]. The experiments were repeated thrice on separate days and the results presented are the averages and the ranges of the three experiments.

### Statistical analysis

The Mann-Whitney test was used to compare the median of IgG subclass and IgE class responses of the INF and PI groups to the antigen. Spearman's rank correlation test was used to test the significance of the correlation between the antibody responses and the age of the patients (expressed as the correlation coefficient, *r*). Unpaired t test with Welch's correction was used to compare mean ± SEM of % L3 molting and % viability in the presence or absence of anti-*Ov*-CPI-2 antibodies and neutrophils. A *p* value of <0.05 was considered significant.

## Results

### Antigenic dominance of *Ov*-CPI-2 in the L3 and mL3 cDNA libraries

A total of 62 L3 and 25 mL3 immunoreactive cDNA clones out of a total of 50,000 and 25,000 PFUs, respectively, were isolated after screening the libraries with a pool of sera from older (>35 years) individuals who were likely to have developed concomitant immunity. The 87 isolated and sequenced clones were grouped into 20 distinctive proteins: 7 in L3, 10 in mL3 and 3 that were present in both L3 and mL3 (Tables 1 and 2). Interestingly, 12 of these larval proteins had already been cloned and characterized using other screening sera and other or similar cDNA libraries. Many of them (*Ov*-CPI-2, *Ov*-RAL-2, *Ov*-FBA-1, *Ov*-103 and *Ov*-73k) had been shown previously to be potentially associated with anti-*O. volvulus* protective immunity [14], [24], or to be potential antigens for the serological diagnosis (*Ov*-16, OV1CF and *Ov*-33) of onchocerciasis (Tables 1 and 2). Moreover, 4 of these (*Ov*-CPI-2 [13], *Ov*-FBA-1 [25], *Ov*-103 [26], [27] and *Ov*-RAL-2 [14], [15], [2] have been proven to be protective in *O. volvulus* mouse model. One of these, onchocystatin (*Ov*-CPI-2), a previously characterized cysteine proteinase inhibitor of *O. volvulus* was the most abundant and second most abundant clone recognized by the immune sera in the L3 (59.7%) and mL3 (16%) cDNA libraries respectively (Tables 1 and 2). The 4 L3s and 4 mL3s novel proteins identified accounted for a total of 10.5% and 16% of the isolated L3 and mL3 clones, respectively. The majority of these *O. volvulus* novel proteins have homologues in *Brugia malayi*. One of the L3 novel proteins is a putative new member of the fatty acid retinoid binding protein family (Accession number GQ202199). This clone, GQ202199, although had only 25% identity to the fatty acid and retinol binding protein-1 of *O. volvulus* (ACL98477.1; *Ov*-FAR-1), it had 60–62% identity to a *B. malayi* putative FAR-1 protein [XP001900470. 1]. We have therefore named this protein *Ov*-FAR-2. Interestingly, *Ov*-FAR-1 (also known as *Ov*-RBP-1 or Ov20) was shown to be also protective in the *O. volvulus* mouse model [14]. Only one encoded protein (Accession number GQ202201) had no homology to the annotated *B. malayi* genome but it had a low level of homology (E value of 3.8; E not significant!) with a hypothetical *C. elegans* protein, B0432.14 (NP_001033326.1).

**Table 1:** cDNA clones isolated from the L3 cDNA library.

SN	Name	GenBank accession	No. of clones	% in screen	% in EST^a^
1	Onchocystatin precursor (cysteine proteinase inhibitor, OV7, Ov-CPI-2), *O. volvulus* [13]	P22085	37	59.7	1.11
2	Fructose 1,6 bisphosphate aldolase of *O. volvulus* (Ov-FBA-1) [25]	AAD38403	6	9.7	0.12
3	OV-17 antigen precursor (Immunodominant hypodermal antigen), *O. volvulus* – Ov-RAL-2 [34]	P36991	4	6.5	0.17
4	Beta-galactoside-binding lectin, *O. volvulus* (Ov-GalBP) [35]	AAA20541	2	3.2	0.14
5	Microfilariae surface-associated protein, *O. volvulus* (Ov-103) [26], [27]	AAA63412	1	1.6	0
6	Gln-rich protein, *O. volvulus* Ov73k [13]	AAC48290	1	1.6	0
7	Cytochrome c oxidase Va polypeptide, with 87% identity to Brugia malayi EDP37396.1; novel and named Ov-COX5a	EU807924.1	1	1.6	0
8	New member of the fatty acid retinoid binding protein with 60–61% identity to Brugia malayi XP_001900470. 1; novel and named Ov-FAR-2	GQ202199	2	3.2	0
9	Troponin T, putative, with 88% identity to Brugia malayi XP_001893395.1; novel and named Ov-TNT-2	GQ202200	1	1.6	0
10	Novel protein with 35% identity to Caenorhabditis elegans NP_001033326.1, but no homologue in B. malayi; novel	GQ202201	1	1.6	0

a , % abundance of clone in the *O. volvulus* EST dataset as reported previously [12].

**Table 2:** cDNA clones isolated from the mL3 cDNA library.

SN	Name	GenBank accession	No. of clones	% in screen	% in EST^a^
1	Ov16, phosphatidylethanolamine binding protein family, *O. volvulus* [36]	P31729	5	20	0
2	Onchocystatin precursor (Cysteine proteinase inhibitor OV7, Ov-CPI-2), *O. volvulus* [13]	P22085	4	16	0
3	Oveg1 (eggshell antigen), *O. volvulus* [37]	AAB35895	3	12	0
4	Ov-17 antigen precursor (Immunodominant hypodermal antigen), *O. volvulus*-Ov-RAL-2 [34]	P36991	2	8	0.17
5	Immunodominant antigen, Ov33-3 precursor	P21250	2	8	0
6	Galectin, *O. volvulus* (Ov87) [38]	AAD00843	2	8	0.28
7	Fructose 1,6 bisphosphate aldolase, *O. volvulus* (Ov-FBA-1) [25]	AAD38403	1	4	0.12
8	Thioredoxin peroxidase, *O. volvulus* (Ov-TPX-2) [39]	AAC48312	1	4	0
9	Intermediate filament, *O. volvulus* (OV1CF) [40]	AAA74283	1	4	0
10	Lactate dehydrogenase, putative, with 95% identity to Brugia malayi EDP30958.1; novel and named Ov-LDH-1	GQ202195	1	4	0
11	EF hand family protein, with 98% identity to B. malayi EDP29905.1 and to troponin c in C. elegans; novel and named Ov-TNC-2	GQ202196	1	4	0
12	Ubiquitin-conjugating enzyme E2-17 kDa, putative with 100% identity to B. malayi EDP37374.1; novel and named Ov-UBC-2	GQ202197	1	4	0
13	Unknown protein, with 99% identity to hypothetical protein of B. malayi, XP_001894199.1; Also Caenorhabditis sp. CB5161 28S rRNA gene (92%); novel	GQ202198	1	4	0

a , % abundance of clone in the *O. volvulus* EST dataset as reported previously [12].

### The development of anti-*Ov*-CPI-2 IgG subclass and IgE class antibody responses in the infected subjects with age

**Figure 1:**
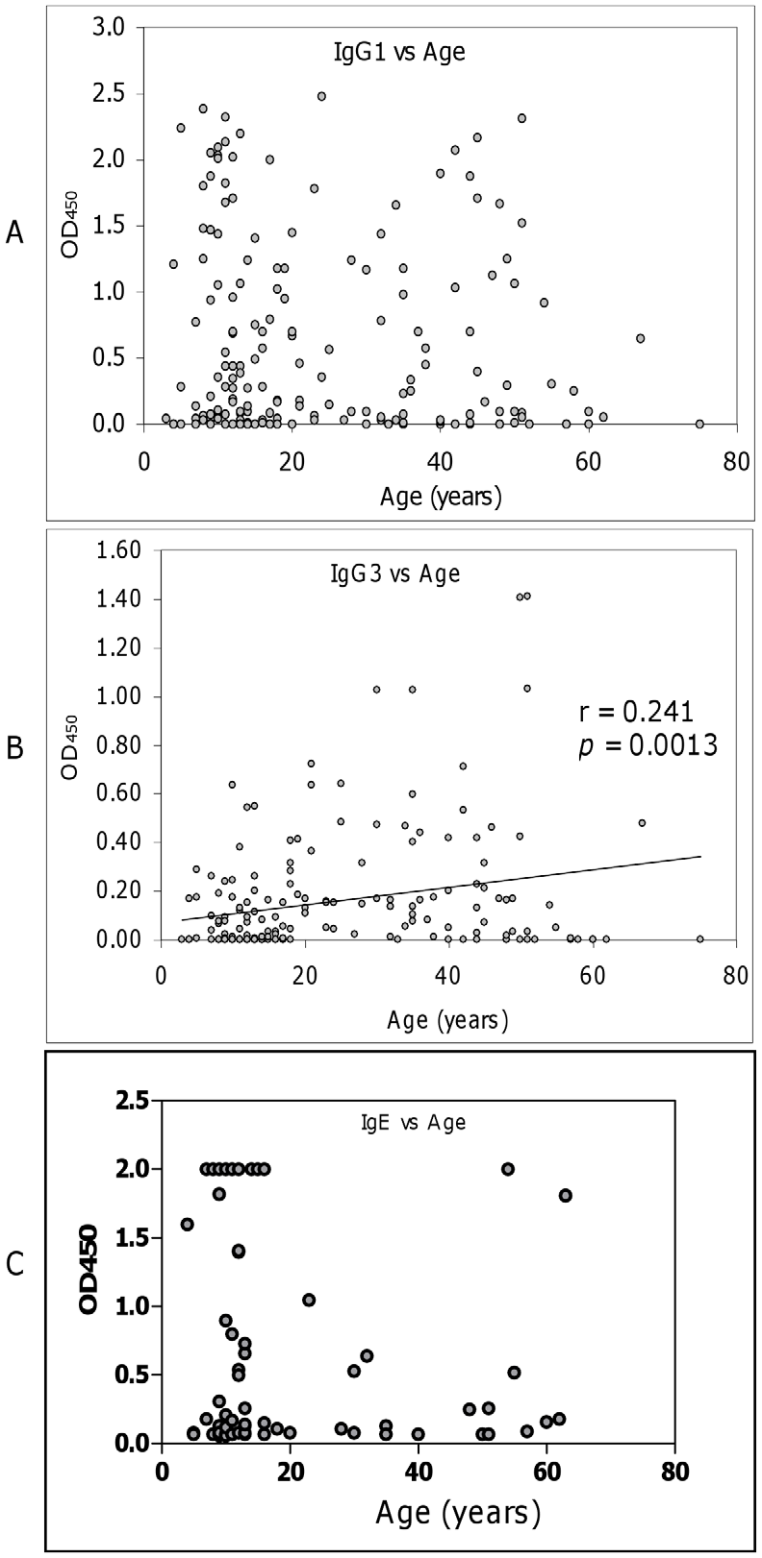
The correlation of IgG sub-classes and IgE class responses to r*Ov*-CPI-2 in infected patients with age: Maintenance of high IgG1 (a) and IgE (c) responses regardless of age; (b) Positive correlation between IgG3 levels and age (r = 0.241; *P* = 0.0013).

Since the recombinant *Ov*-CPI-2 (r*Ov*-CPI-2) protein was identified by immunoscreening with sera from infected individuals 35 years of age or more who are likely to have developed concomitant immunity, it was of interest to investigate the development of the IgG1, IgG3 and IgE antibody responses to the antigen in infected individuals in relation to their age (N = 176 for IgG1 and IgG3 analyses; N = 68 for IgE analysis). Although the IgG1 responses to r*Ov*-CPI-2 antigen were not correlated with age (Fig. 1a), they were relatively elevated in all ages; with an overall 82.4% of IgG1 responders (mean 0.61±0.72). For the IgG1 analysis, there was no significant difference also in the proportion of responders vs. the non-responders in individuals of ≤20 years of age or those >20 years of age. The IgG3 response to the r*Ov*-CPI-2 antigen was, however, positively correlated with age (r = 0.241; *p*<0.01) (Fig. 1b). For the IgE responses there was no significant correlation with age (Fig. 1c).

### Immune responses to rOv-CPI-2 in the putatively immune individuals vs. the *O. volvulus* infected subjects

**Figure 2:**
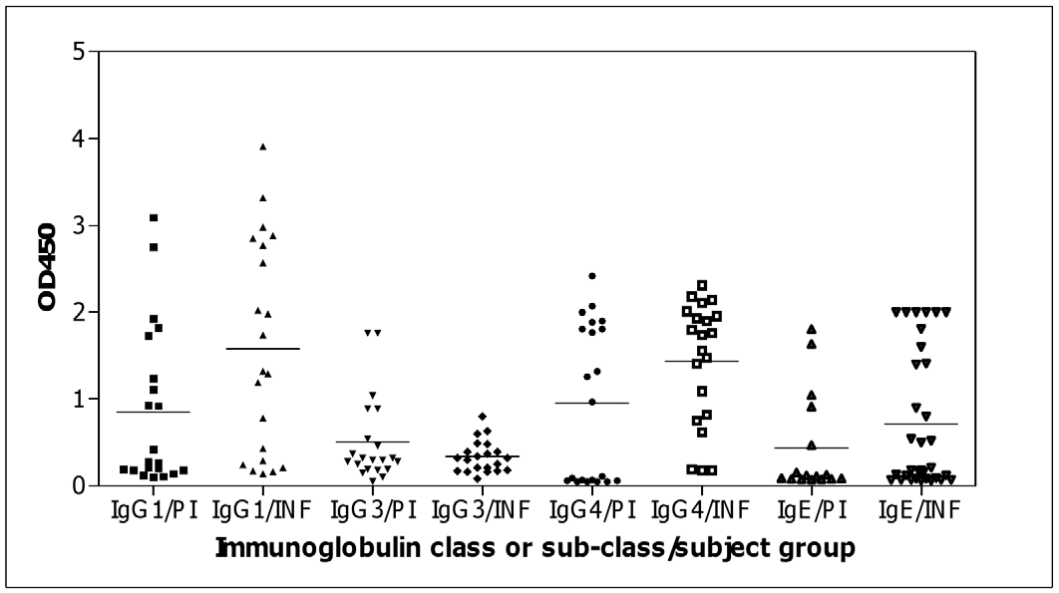
Human IgG sub-classes and IgE class antibody responses to recombinant *Ov*-CPI-2 in the PI and the INF. The median IgG1, IgG3, IgG4 and IgE responses to r*Ov*-CPI-2 in the PI (N = 21) and INF subjects (N = 21) are indicated with short, solid horizontal lines. The median IgG1 and IgG4 responses are significantly higher in the INF (*P* = 0.03 and 0.04 respectively) than in the PI.

Since the r*Ov*-CPI-2 protein was identified by immunoscreening with sera from infected individuals who are likely to have developed concomitant immunity, it was of interest to also investigate whether the native protein also induced the production of IgG and IgE antibodies in humans categorized as putatively immune (PI). These individuals have been exposed to the parasite over several years but have not developed clinical or parasitological signs of infection. The IgG subclass responses to r*Ov*-CPI-2 in the putatively immune individuals (N = 21) were analyzed in comparison to the responses in age- and sex-matched infected individuals (N = 21). As shown in Figure 2, although the PI and INF individuals had similar anti-r*Ov*-CPI-2 specific IgG3 responses (median of 0.3 and 0.32, respectively), the PI individual serum samples were generally more reactive with the antigen than the INF individuals (Upper 95% CI value of 0.730 for PI vs. 0.417 for INF). The median IgG1 responses of the INF, however, were significantly higher (*p* = 0.03) than those of PI (Fig. 2). The IgG4 responses against r*Ov*-CPI-2 were also significantly higher (*p* = 0.04) in the INF than in PI as expected since IgG4 antibody responses are generally associated with an active and patent infection. On the other hand, the IgE responses between the two groups were not significantly different.

### Cytotoxic effect of anti-Ov-CPI-2 antibodies on L3 larvae in the presence of normal neutrophils

*In vitro* cytotoxicity assays demonstrated that purified monospecific human antibodies against r*Ov*-CPI-2 in the presence of normal human neutrophils were able to inhibit 91% of molting from L3 to L4 as determined on day 6 in culture (Table 3), and resulted in almost a total loss of larval viability (72%). In normal conditions, about 43–68% of L3s molt to L4 in 6 days. The differences in % molting between the complete medium control and the negative non-specific antibodies control were not statistically different, while they are highly significant when the effect of monospecific anti-*Ov*-CPI-2 antibodies on molting was compared with the two controls (*P* = 0.0007 and *P* = 0.028 respectively). However, the viability of the L3 that did not molt in the presence of anti-*Ov*-CPI-2 antibodies was not statistically different from that of the control cultures. This was mostly due to the fact that in one of the three experiments the % viability was 82% vs. 0% and 2% in the other two experiments (data not shown). Longer cultures up to 10 days might be needed for consistent killing data.

**Table 3:** Effect of mono-specific human anti-*Ov*-CPI-2 antibodies on L3 molting and viability in the presence of normal human neutrophils.

	% Molting^a^		Inhibition of molting		% Viability of larvae	
	Mean ± SEM	Range	%	Range	Mean ± SEM	Range
Medium control	64±3.0	58−68	—	—	94.6±1.3	92−96
Negative antibodies	50.6±13.3	43−66	20.9	12−34	78.7±8.7	65−95
Anti-Ov-CPI-2 antibodies	3.7±2.7*	0−9	94.2	79−100	28±27.0**	0−82

a *O. volvulus* L3 were cultured in complete medium alone or in complete medium containing negative control antibodies purified from the same serum donor using a λgt11 lysate column or mono-specific antibodies to r*Ov*-CPI-2 in the presence of 2×10^5^ normal human neutrophils per well for 6 days. Larval molting and viability were determined as described under the “Materials & Methods” section. Three experiments were performed, where each consisted of a total of 10 wells per treatment group with 5 larvae per well. The results are the mean ± SEM and the range of the three experiments.

*Significance between groups was determined by unpaired t test with Welch's correction with *p* = 0.007 between medium control and the anti-*Ov*-CPI-2 group and *p* = 0.028 between the negative antibodies and the anti-*Ov*-CPI-2 group.

**There is no significant difference between the three groups.

## Discussion

The objective of the present study was to identify *O. volvulus* larval antigens that may be important in the development of concomitant immunity to the L3 that develops in the INF with increasing age, and which is independent of the immune responses that are directed against the adult worms and microfilariae associated with patent infection [6]. Concomitant immunity is a concept initially developed in cancer immunology and later also in lymphatic filariasis [5], [9]. It was only recently reported in onchocerciasis, where it is believed to contribute in preventing most of the newly acquired L3 infections from developing, and results in a stabilization of the adult worm and microfilarial burdens with age in the INF [3], [6], [4]. Thus, a pool of sera from individuals who have likely developed such immunity, based on their high IgG1 and IgG3 responses to crude extracts of L3 and mL3, was used to screen the corresponding cDNA libraries. The 20 distinct immunoreactive antigens identified included 4 proteins (*Ov*-CPI-2, *Ov*-FBA-1, *Ov*-103 and *Ov*-RAL-2) that had previously been shown to be protective in a small animal model [13], [25], [26], [27], [14], [15], [2].

*Ov*-CPI-2 (*O. volvulus* cystatin, also known as onchocystatin) appeared to be, overall, the most immunodominant cDNA clone reacting with the pool of sera. The high frequency of detection of the *Ov-cpi-2* clone in the present study may be a reflection of its abundance in the L3 EST datasets as reported previously [12] and/or of the particular mix of antibodies against the larval proteins in the pooled sera we used for screening. Due to its immunodominance, *Ov*-CPI-2 was selected for further characterization with a focus on its increased recognition with age, a phenomenon that has been previously associated with the development of concomitant immunity [6] and thus strengthening its selection as a promising subunit vaccine candidate against *O. volvulus* infection. *Ov-*CPI-2 was previously cloned using antisera from chimpanzees experimentally immunized with attenuated L3s [28]. It was shown to be also expressed in all the other stages of the *O. volvulus* parasite except in mature microfilariae [28]. It was localized in the hypodermis, basal layer of the cuticle and in the eggshell of developing microfilariae [28]. Further studies established that this 17 kDa protein could be essential for development of the infective stage larvae as it probably regulates endogenous cysteine proteases that are essential for molting [29], [30]. Subsequent vaccination-challenge experiments in *O. volvulus* mouse model using recombinant GST-*Ov*-CPI-2 and alum as the adjuvant showed that it could induce significant protection levels of 43–49% [13]. Further support for its protective role has come from protective studies done with its homologues in other nematodes and including lymphatic filariae [31], [32], [33].

Analysis of the individual levels of the cytophilic antibody IgG3 to *Ov*-CPI-2 as a function of age showed an age-dependent association of the antibody responses, which were significantly increased with age and therefore consistent with the development of concomitant immunity to larval antigens in this population [6]. The INF and the PI individuals had similar anti-r*Ov*-CPI-2 specific IgG3 responses (median of 0.3 and 0.32, respectively), while the INF had clearly higher IgG1 and IgE responses. The maintenance or up-regulation of cytophilic (IgG1, IgG3, and IgE) and complement-fixing (IgG3) antibody responses against larval antigens, some of which are not necessarily larvae specific, as was found in this study was shown before to be also associated with immune protection in onchocerciasis [6], [16], [17].

Our results provide further support to the possibility that the anti-*Ov*-CPI-2 antibodies may have a role in the ADCC effector mechanisms. We observed that human neutrophils inhibited molting of L3 by 79–100% in the presence of purified human mono-specific antibodies against *Ov*-CPI-2. Johnson et al [21] have shown that sera from PI and INF are able to inhibit molting of L3 and kill them when cultured in the presence of neutrophils from normal humans. Antibodies and ADCC were also found to be an important part of protective effector mechanisms in *O. volvulus* mouse model [15], [2].

In one preliminary study, when r*Ov*-CPI-2 was used to stimulate human peripheral blood mononuclear cells (PBMCs) from INF individuals (n = 6) and PI (n = 4), it appeared that it induced a mixed IFN-γ and IL-5 response (cytokine levels were determined by ELISA) in approximately 50% of the samples (data not shown). When the cytokine responses were determined using the ELISPOT assay and PBMCs from additional individuals, 6 out of 6 PI individuals had higher (∼10×) frequencies of IFN-γ than IL-5-producing cells, while in the INF group 8 out of 10 individuals had IL-5 dominated responses, and the remaining 2 were only IFN-γ responders. Although the numbers tested so far are small, it points to the possibility that in the PI, the Th1 cytokine response dominates, while a more mixed Th1/Th2 response against *Ov*-CPI-2 is observed in the INF individuals. Overall, the data presented in this study clearly support the notion that *Ov*-CPI-2 is a promising leading target of protective immunity in onchocerciasis and the results discussed justify further vaccination studies on this antigen for human use.
